# Acute tubulointerstitial nephritis with germinal centers in antineutrophil cytoplasmic antibody-associated vasculitis: A case report and literature review

**DOI:** 10.1097/MD.0000000000018178

**Published:** 2019-11-27

**Authors:** Zi-Shan Lin, Xiao-Ling Liu, Zhao Cui, Su-Xia Wang, Feng Yu, Fu-De Zhou, Ming-Hui Zhao

**Affiliations:** a Renal Division, Department of Medicine, Peking University First Hospital; Institute of Nephrology, Peking University; Renal Pathology Center, Institute of Nephrology, Peking University; Key Laboratory of Renal Disease, Ministry of Health of China; Key Laboratory of CKD Prevention and Treatment, Ministry of Education of China; b Laboratory of Electron Microscopy, Pathological Centre, Peking University First Hospital; c Department of Nephrology, Peking University International Hospital; d Peking-Tsinghua Center for Life Sciences, Beijing 100034, P.R. China.

**Keywords:** anti-mCRP antibody, antineutrophil cytoplasmic antibody-associated vasculitis, germinal center, rapidly progressive glomerulonephritis, tubulointerstitial nephritis

## Abstract

**Rationale:**

Occasionally, tubulointerstitial lesions can be found in antineutrophil cytoplasmic antibody (ANCA)-associated vasculitis (AAV). However, significantly isolated tubulointerstitial nephritis (TIN) with germinal centers is rare.

**Patient concerns:**

A 17-year-old Chinese Han patient showed rapidly progressive glomerulonephritis, anuria, and serum creatinine of 19.4 mg/dL.

**Diagnosis:**

He had positive ANCA targeting myeloperoxidase (55.0 RU/mL). The renal biopsy showed crescent formation in 100% of glomeruli. Of special note, the glomerular crescents were surrounded by granulomatous inflammation, extensive tubular destruction or disappearance, and massive interstitial infiltration. A diagnosis of AAV was thus made with the involved organ restricted to the kidney.

**Interventions:**

The patient underwent 7 rounds of plasmapheresis, 3 pulses of methylprednisolone therapy (500 mg per pulse), and oral prednisolone (50 mg/d). Rituximab (500 mg) was used after the plasma exchange treatment.

**Outcomes:**

ANCA was negative, while anti-modified C-reactive protein (anti-mCRP) antibodies remained positive. The patient was dependent on hemodialysis. We found anti-mCRP antibody in the serum of the patient, with the major epitope on amino acids 35 to 47 of mCRP.

**Lessons:**

We proposed that the anti-mCRP antibody might play an important role in this case of acute TIN in AAV.

## 1 Introduction

Renal involvement is common in antineutrophil cytoplasmic antibody (ANCA)-associated vasculitis (AAV), and patients often present with pauci-immune necrotizing crescentic glomerulonephritis.^[1]^ Tubulointerstitial (TI) lesions can also be found in the kidneys in AAV, although their pathogenesis remains to be elucidated.^[1–3]^ Modified C-reactive protein (mCRP) is a tissue and/or cell-based form of the acute phase protein and has been suggested to be a possible antigen in acute tubulointerstitial nephritis (ATIN). However, there is no report of an anti-mCRP antibody in patients with AAV. Here, we present a case of ATIN in AAV with positive serum anti-mCRP antibody, which might provide some insights into the pathogenesis of the disease.

## 2 Case presentation

A 17-year-old Chinese Han man was admitted with a 23-day history of edema, fatigue, and subsequent anuria. He had no fever. Ten days before admission, his serum creatinine was 19.4 mg/dL, and urinalysis revealed proteinuria 1+ and hematuria with 228 red blood cells/high-power field (HPF). He was positive for perinuclear ANCA (pANCA) by immunofluorescence. He received hemodialysis and renal biopsy and was referred to our hospital. He had suffered from hyperthyroidism for 7 years and had been prescribed thiamazole and propylthiouracil (PTU) for 3 years. Moreover, he had a 5-year history of allergic rhinitis and allergic asthma. He had no family history of hypertension, glomerulonephritis or end-stage renal disease, and he had no drug addiction.

On admission, the physical examination revealed a blood pressure of 128/73 mm Hg, temperature of 36.5°C, heart rate of 78/min, and respiratory rate of 20/min. The patient was anemic, although further systemic clinical examination was unremarkable.

The laboratory data revealed serum creatinine of 12.3 mg/dL and interleukin-6 of 66.0 pg/mL (normal range: 0–0.64 pg/mL). The patient's urinalysis revealed proteinuria 1+ and dysmorphic red blood cells >100/HPF; he was positive for pANCA by immunofluorescence, and anti-MPO antibody was shown to be positive at 55.0 RU/mL by enzyme-linked immunosorbent assay. Anti-glomerular basement membrane antibody and antinuclear antibody were negative. The ophthalmological examination excluded tubulointerstitial nephritis with uveitis (TINU), and positron emission tomography-computed tomography scan indicated no tumor or infection.

**Figure 1:**
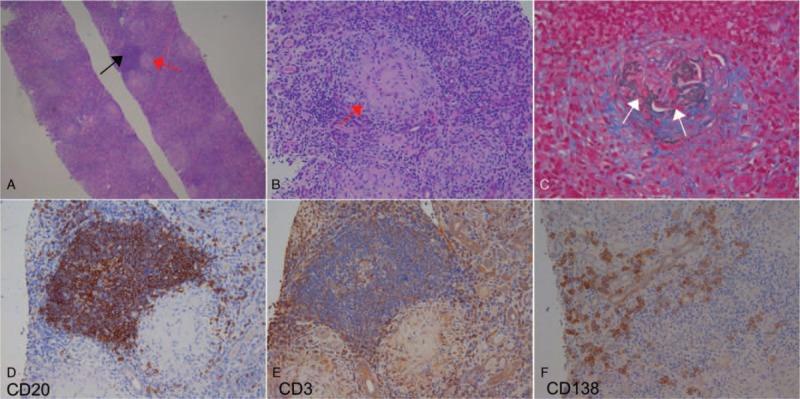
The pathological findings of renal biopsy. (A) The renal parenchyma was diffusely destroyed, all glomeruli exhibited crescents, and Bowman capsules were disrupted and surrounded by a granuloma (red arrow), along with tubular atrophy and disappearance, massive interstitial infiltration of inflammatory cells, and the “germinal center” (black arrow) formation in the interstitium (HE ×40). (B) The granuloma (red arrow) showed the destruction of the glomerular structure and the disrupted Bowman capsule (HE ×200). (C) Disruption of the glomerular basement membrane (white arrows) surrounded by a granuloma (PASM + Masson ×200). Of one “germinal center” in the interstitium, CD20+ B cells (D) were clustered in the middle area and surrounded by CD3+ T cells (E). Scattered CD138+ plasma cells (F) were observed in the surrounding parenchyma (D–F, ×200).

The renal histology showed diffuse destruction of glomerular structure with crescents and severely ruptured Bowman capsules (Fig. 1), which were surrounded by granulomatous inflammation, massive destruction or disappearance of tubules, and extensive interstitial infiltration of lymphocytes, monocytes, eosinophils, and plasma cells, with focal lymphocyte aggregation into “germinal centers” (Fig. 1). Immunofluorescence microscopy showed little, if any, deposition of immunoglobulin or complement in the glomeruli and tubulointerstitium. Immunohistochemical staining (Fig. 1) showed that the interstitial infiltrated cells were positive for CD20 (++), CD3 (+), CD138 (++), and Bcl2 (+), and negative for CD23, CyclinD1 and CD30.

**Figure 2:**
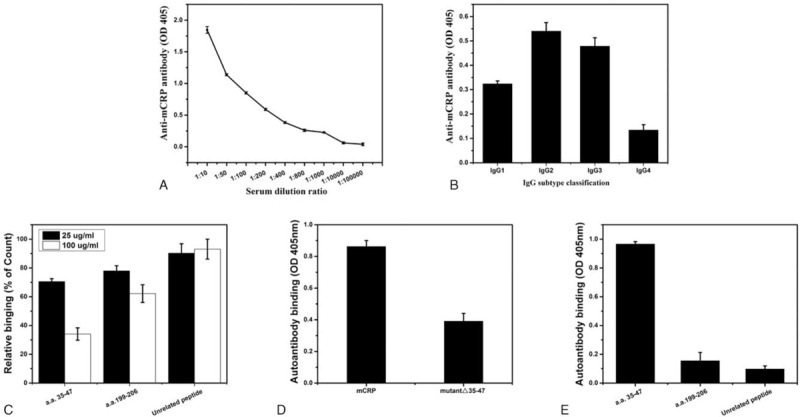
The anti-mCRP antibodies in the patient. (A) Titer, 1:800. (B) IgG subclass, IgG2, and IgG3. (C–E) Epitope, a.a. 35 to 47. (C) The serum of the patient was mixed with the indicated peptides and added to immobilized mCRP; a.a. 35 to 47 was the only peptide that significantly reduced the binding between mCRP and its antibodies. (D) mCRP and mutant mCRP lacking a.a. 35 to 47 (mutant Δ35–47) were expressed in *Escherichia coli* and purified, and their binding to immobilized anti-mCRP antibodies was examined. mCRP showed strong binding, whereas the binding capacity was lost upon deletion of a.a. 35 to 47. (E) Synthesized CRP peptides were immobilized and tested for binding to antibodies from this patient. Apparent binding to a.a. 35–47 was observed. a.a. = amini acids, IgG = immunoglobulin G, mCRP = modified C-reactive protein.

Because of the severe TI injury observed in his kidney specimens, we detected anti-mCRP antibodies. The patient was strongly positive (112%) for serum anti-mCRP antibodies. As illustrated in Figure 2, the anti-mCRP antibody titer was 1:800, the subclasses of immunoglobulin G (IgG) were IgG2 and IgG3, and the major epitope was amino acids (a.a.) 35 to 47. Eight months later, the titer was still 1:800, while the main IgG subclass was IgG1.

The patient received 7 rounds of plasmapheresis, 3 pulses of methylprednisolone therapy (500 mg per pulse), and oral prednisolone (50 mg/d). Rituximab (500 mg) was used after the plasma exchange treatment. ANCA was negative, while anti-mCRP antibodies remained positive. The patient was dependent on hemodialysis.

## 3 Discussion

This patient presented with rapidly progressive glomerulonephritis, positive serum ANCA and pauci-immune crescentic glomerulonephritis with severe interstitial nephritis. Thus, a diagnosis of AAV was highly suspected. However, several other diseases should be excluded.

First, PTU-induced ANCA-positive vasculitis must be highly suspected. Increasing evidence has demonstrated that PTU could induce ANCA-positive vasculitis, and the withdrawal of the drug was closely related to the improvement of active vasculitis and renal function.^[4]^ However, this patient stopped taking PTU 4 years ago. Second, TINU syndrome also need to be considered. However, his ophthalmological examination was normal, and severe glomerular crescent formation was not consistent with the characteristics of TINU.

By excluding other possible diagnoses, a diagnosis of AAV was made with its target organ restricted to the kidney.

Renal involvement of AAV is predominant in glomerular lesions, especially pauci-immune necrotizing crescentic glomerulonephritis. The TI lesions are thought to be secondary damage to the glomeruli.^[1–3]^ However, the current patient showed severe TI lesions with germinal centers and Bowman capsule rupture, which could not be explained simply by AAV.

To our knowledge, there are 16 reported cases of TIN with positive ANCA (Table 1).^[5–15]^ In some cases, renal function improved with decreased ANCA levels after immunosuppressive therapy. The mechanism of isolated TIN in AAV remains to be clarified. Sato et al^[16]^ and Nakabayashi et al^[5]^ proposed that peritubular capillary injury was the pathogenesis of the TI changes in AAV, and the latter suggested that early loss of CD34 antigenicity in the peritubular capillary played an important role. Kasahara et al^[6]^ proposed that low-affinity MPO-ANCA may recognize some antigens specific to the TI area. Nakamura et al^[7]^ and Hassani et al^[8]^ proposed that endothelial tubular cell injury may be induced by the adhesion of leukocytes that express MPO and proteinase 3 on the cell surface. Banerjee et al^[9]^ and Wen et al^[10]^ proposed that activated neutrophils in the renal interstitium could contribute to a direct cell-mediated injury to the interstitium.

**Table 1:**
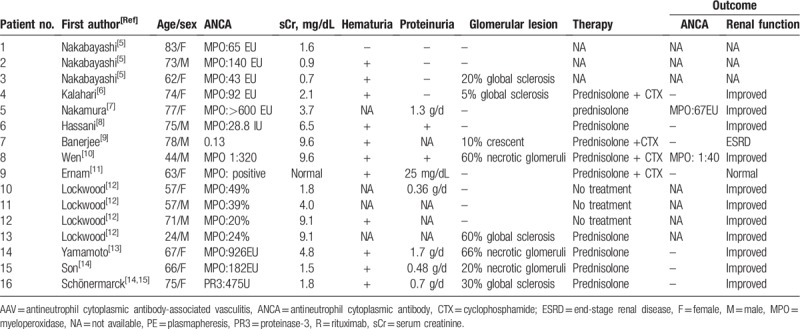
Summary of the reported case with tubulointerstitial nephritis with AAV.

mCRP is a tissue and/or cell-based form of the acute phase protein. Our previous study found that mCRP might be a target autoantigen in TINU syndrome,^[17]^ which is characterized by TIN with bilateral sudden-onset anterior uveitis. The circulating level of anti-mCRP autoantibodies in patients with lupus nephritis (LN) was closely associated with the score of their interstitial lesions.^[18]^ Recently, Li et al^[19]^ showed that a.a. 35 to 47, a sequence exposed only in mCRP, constitutes the major epitope recognized by anti-CRP autoantibodies in patients with LN and indicates that mCRP binds complement factor H and enhances its cofactor activity via a.a. 35 to 47, whereas autoantibodies against this epitope inhibit these actions and adversely affect LN. The anti-mCRP autoantibody titer in this patient was high, and its IgG3 subclass is pathogenic. Furthermore, its epitope is a.a. 35 to 47. Thus, we suggest that anti-mCRP antibodies might play an important role in the patient's interstitial lesions.

More interestingly, there were many ectopic germinal centers and nonlymphoid collections of mature B lymphocytes in the renal interstitium of this patient, suggesting that severe renal interstitial inflammation may be associated with his autoimmune disease. The patient also presented with elevated interleukin (IL)-6. Recently, Espeli et al^[20]^ showed that in LN, the kidneys are a major source of autoantibody-producing plasma cells. The inflammatory cytokine IL-6 supports long-lived plasma cell survival in vitro and is increased in the serum of patients with lupus. Arkatkar et al^[21]^ demonstrated that B cell-derived IL-6 is critical for spontaneous germinal center formation. Ectopic germinal center formation may contribute to the progression of lupus interstitial nephritis by selecting for cells that locally secrete pathogenic antibodies in the tubulointerstitium.^[22]^ It is unclear whether a similar process occurred in the current case, but further study is needed.

In conclusion, we reported a case of severe TI lesions with many germinal centers and rupture of all Bowman capsules in AAV, which may result from anti-mCRP antibodies.

## 4 Statement

The study was performed in compliance with the Declaration of Helsinki and approved by the Ethics Committee of Peking University First Hospital. Informed written consent was obtained from the patient for publication of this case report and accompanying images.

## Author contributions

**Methodology:** Zi-Shan Lin, Xiao-Ling Liu.

**Supervision:** Zi-Shan Lin.

**Validation:** Zi-Shan Lin.

**Visualization:** Zi-Shan Lin.

**Writing – original draft:** Zi-Shan Lin.

**Writing – review and editing:** Zhao Cui, Su-Xia Wang, Feng Yu, Fu-De Zhou, Ming-Hui Zhao.
